# Supplementary material for: Causal interplay between lactose intolerance and gut microbiota: a combined bidirectional Mendelian randomization and in vivo validation study
Source: Front Nutr. 2026 Jun 1;13:1803337. doi: 10.3389/fnut.2026.1803337 (PMC13265576; doi:10.3389/fnut.2026.1803337)
Supplement: Supplementary file 1 [file Data_Sheet_1.zip › supplementary materials/Forward/leave one out cross validation/ebi-a-GCST90027817.finngen_R12_E4_LACTONAS.pdf]

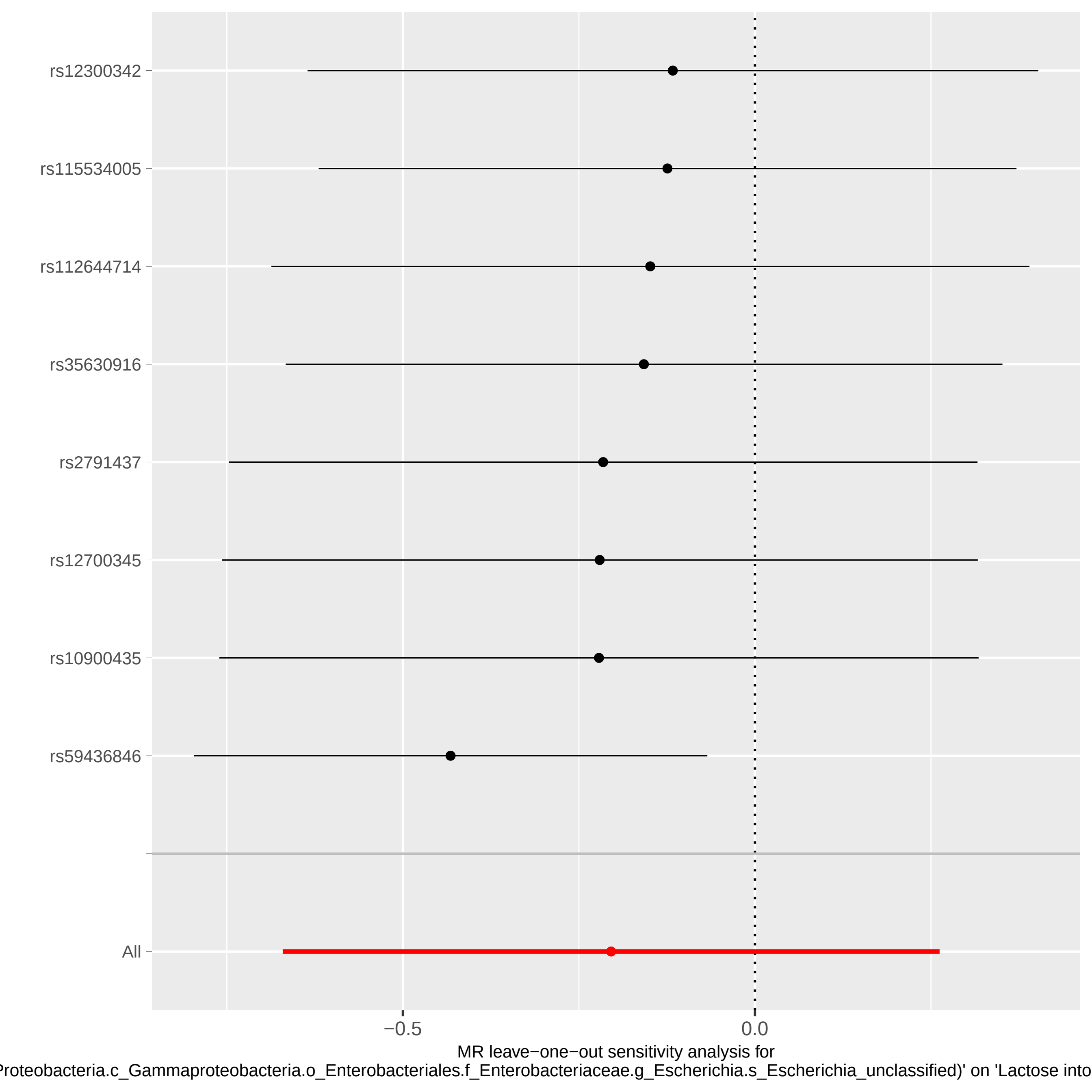

rs12300342
rs115534005
rs112644714
rs35630916
rs2791437
rs12700345
rs10900435
rs59436846
All
−0.5
0.0
MR leave−one−out sensitivity analysis for
Proteobacteria.c_Gammaproteobacteria.o_Enterobacteriales.f_Enterobacteriaceae.g_Escherichia.s_Escherichia_unclassified)' on 'Lactose into
